# Perceived Stress in Hepatitis C Virus Infected Patients under the DAA-Based Therapy

**DOI:** 10.3390/diagnostics12051177

**Published:** 2022-05-09

**Authors:** Claudia Monica Danilescu, Mihaela Ionescu, Daniela Larisa Sandulescu, Mihail Cristian Pirlog, Costin Teodor Streba, Ion Rogoveanu

**Affiliations:** 1 Doctoral School, University of Medicine and Pharmacy Craiova, 200349 Craiova, Romania; monica.danilescu@gmail.com; 2 Department of Medical Informatics and Biostatistics, Faculty of Medicine, University of Medicine and Pharmacy of Craiova, 200349 Craiova, Romania; mihaela.ionescu@umfcv.ro; 3 Department of Gastroenterology, Faculty of Medicine, University of Medicine and Pharmacy of Craiova, 200349 Craiova, Romania; ionirogoveanu@gmail.com; 4 Department of Medical Sociology, Faculty of Medicine, University of Medicine and Pharmacy of Craiova, 200349 Craiova, Romania; 5 Department of Scientific Research Methodology, Faculty of Medicine, University of Medicine and Pharmacy of Craiova, 200349 Craiova, Romania; costinstreba@gmail.com

**Keywords:** hepatitis C virus, direct acting antivirals, perceived stress, fibrosis, sustained virological response

## Abstract

The Hepatitis C Virus (HCV) infection often associates medical and mental health conditions which lead to increased levels of distress. Our study aimed at assessing the level of perceived stress on a sample of 90 HCV infected patients treated with Direct-Acting Antiviral (DAA) agents for 12 weeks, and its possible correlations with clinical and evolutionary elements. The evaluation was conducted in three phases: before administration of the DAAs (BSL), at the End of the Treatment (EOT), and 24 weeks after the BSL (Sustained Viral Response—SVR). The perceived stress was measured using the Perceived Stress Scale (PSS). The efficiency of the DAA treatment reduced the levels of stress (98.99% moderate and high stress at BSL to 70.00% at SVR). It was observed, for the entire study period (BSL to SVR), that the decrease in the perceived stress severity was significantly associated with demographic items such as gender (*p* < 0.01), urban environment (*p* < 0.001), the age of the subjects (*p* < 0.05), and clinical data such as F4 degree of fibrosis (*p* = 0.001) and overweight or obesity class II (*p* < 0.01). The perceived stress is directly associated with the severity of the HCV infection, and it could be significantly lowered by an efficient therapeutic approach, as DAAs are nowadays.

## 1. Introduction

Since it was discovered, infection with the Hepatitis C Virus (HCV) has represented a complex challenge for both affected individuals and clinicians. The specific symptoms of the disease are often accompanied by mental health conditions [1,2,3], poorer quality of life and financial losses [4,5], disturbed social statuses [6,7], and increased healthcare needs [8,9], all leading to increased levels of distress [10,11].

The way in which each of the patients with HCV infection perceives the action of stressors depends on individual characteristics: the evolution of the liver disease and the degree of symptoms severity, respectively, as well as the personal resilience and development of coping mechanisms.

Selye postulated, almost one hundred years ago, “Everyone knows what stress is, but nobody really knows”, and also gave a generic definition of stress as “the nonspecific response of the body to any demand” [12]. The most common effects of stress are anxiety, fear, depression, and post-traumatic stress disorder (PTSD), and it could lead to the fight or flight response [13]. Considering that HCV infection as a severe medical condition, the presence of emotional distress is the consequence of multiple issues, including physical symptoms produced by the disease and by the associated treatment. Moreover, nonspecific symptoms (e.g., asthenia, general malaise, migraine, muscular pain, pruritus) may also be present, and could qualify as triggers for psychological problems [14]. Other contributors to stress are the individual ways in which the disease’s symptoms are perceived, i.e., the negative effects on the physical and psychological well-being and the disturbance of social functions [15].

There is evidence that emotional distress is present in at least 35% of the HCV infected patients, with a peak of 71% in those with a diagnosis of HCV and active psychiatric or medical comorbidities. When comorbidities were not reported, 20% of the affected individuals had a degree of stress which was double that of the general population [10].

The magnitude of this emotional distress is directly influenced by the individuals’ perception of stress and their ability to develop coping mechanisms toward the stressors. When the patients are not able to create proper strategies to deal with stressors, the impact of the emotional disturbances could not only lead to a worsened evolution of the liver disease, but also to a higher severity of the psychological issues. For example, patients who faced many medical interventions and hospital admissions had significantly higher emotional distress than newly diagnosed patients [10]. This emphasizes the importance of the medical process itself in the emotional management of these patients.

As mentioned above, an important source of stress is represented by the side-effects of the medication, especially if previous generations of HCV pharmacological standard care (pegylated Interferon) [16,17] are considered. Nowadays, new therapeutic tools represented by Directly Acting Antivirals (DAAs) have less psychological side-effects and a better efficiency and safety profile. Under these circumstances, it could be hypothesized that the level of perceived stress is also lowered by the consecutive effects on the patient’s state of well-being. Starting from this hypothesis, the aim of our study was to assess the level of perceived stress on a sample of HCV infected patients and possible correlations with clinical and evolutionary aspects.

## 2. Materials and Methods

During a seventeen-month period (August 2017–December 2018), we conducted a prospective study on a sample consisting of 90 individuals diagnosed with the HCV infection. The subjects of the study were outpatients under the treatment and monitoring process in the Gastroenterology Clinic of the Emergency Hospital in Craiova, Dolj County, Romania. All received pharmacological therapy with DAAs: Ombitasvirum/Paritaprevirum/Ritonavirum/Dasabuvirum combinations for a 12-week period, in accordance with the guidelines provided by the Romanian Society of Gastroenterology and Hepatology (RSGH) [18,19], the European Association for the Study of the Liver [20], and the Romanian National Protocol for HCV infected patients (no. 280, code J05AP) (2017) [21].

The subjects were initially selected to fulfill the criteria to be included in the therapeutic Romanian national program of interferon-free therapy [21]. In order to be included in the program, the individuals were clinically and biologically assessed (screening visit) and their eligibility was confirmed. For our study purposes, we have added to these inclusion criteria the following: acceptance to participate based on signed informed consent and absence of any diagnosed and treated neurologic and/or psychiatric condition in the last 12 months (Table 1).

All of those who qualified for our study were rechecked at the first study visit (baseline—before the initiation of the DAA treatment—BSL), both on the current clinical status and on personal medical history by the gastroenterologist, through a structured clinical interview. The subjects’ gender, age, residence, height, weight, the virus’ route of transmission, Body-Mass-Index—BMI, fibrosis degree measured by the FibroTest^®^ and/or with FibroScan^®^ methods (F0, F1, F2, F3 or F4), level of the HCV ribonucleic acid viral load (HCV-RNA) and alpha-fetoprotein (AFP) (IU/mL), alanine aminotransferase (ALT) (IU/L), aspartate aminotransferase (AST) (IU/L), and the level of perceived stress were recorded. The evaluation of the route of transmission has shown that all subjects were not illegal drug users, which is consistent with the results of epidemiological studies of HCV infection in Romania [22].

The next evaluation was conducted at the end of the DAA treatment period (12 weeks after baseline—EOT) and included the same procedures as at BSL.

Finally, the third assessment point was at the follow-up (Sustain Viral Response—12 weeks after the end of the treatment—SVR) when same package of clinical and laboratory data was assessed [3].

The level of the perceived stress was evaluated using the Perceived Stress Scale (PSS), one of the most reliable and popular stress assessment instruments [23]. Our study was based on the original 14-item version of the PSS, including 7 positive items and 7 negative items, rated on a 5-point Likert scale ranging from 0 = ‘never’ to 4 = ‘very often’. The total score is the amount of each item’s rating, ranging from 0 to 56, and describes the overall level of perceived stress, with the following cut-off values: 0–13 (low stress), 14–26 (moderate stress), 27–40 (high perceived stress) [23]. This self-reported questionnaire was designed to measure the degree to which situations in one’s life were appraised as stressful over the previous months. The PSS-14 proved established acceptable psychometric properties, with good internal consistency reliability (Cronbach’s alpha >0.70) and test–retest reliability (Pearson’s, Spearman’s, or the intraclass correlation >0.70) [24], with similar data obtained over its Romanian version [25,26,27].

The questionnaire’s purpose was explained by the gastroenterologist and the research assistant and then filled in by the patients, in a pen–paper way, during the visits at all the three assessments (BSL, EOT, SVR). The obtained data were recorded and secured in a dedicated digital database.

Due to the fact that all subjects were actually patients included in the Romanian National Program for HCV interferon-free therapy, we did not record any drop-outs during the study period. More than that, the specific visits for our research coincided with the national program’s timetable. All subjects were included on a voluntary basis and signed the informed consent form. The study was approved by the Ethics Committee of Craiova University of Medicine and Pharmacy of Craiova and was in line with the Helsinki Declaration.

### Statistical Analysis

The study database included all clinical and non-clinical items and results of the applied questionnaires, all recorded in a Microsoft Excel file. For the descriptive analysis, continuous variables were expressed in terms of absolute and relative frequencies (%) and mean ± standard deviation. The analysis of the possible correlations between these was performed using Statistical Package for Social Sciences (SPSS), version 20 (IBM Corp., New York, NY, USA) and the following statistical tools: Kendall’s tau-b correlation, Spearman’s rank-order correlation, Friedman test for repeated measures, Shapiro–Wilk’s test for data normality analysis, Levene’s test of equality of variances, and Mann–Whitney U and Kruskal-Wallis H tests to determine whether there were differences between groups. Chi-square test was used for categorical data (χ^2^). For the purposes of the present study, the following *p* values were accepted: *p* < 0.05 significant in a confidence interval (CI) of 95%, *p* < 0.01 (CI of 99%), *p* < 0.001 highly significant (CI of 99.9%).

The power of the study based on its sample size was calculated according to the G*Power 3.1.9.7 methodology (Heinrich Heine University Düsseldorf, Düsseldorf, Germany). Hence, a minimum sample size of 42 subjects (49 subjects for a 15% correction) was suggested. This calculation was based on a Kendall’s W as an effect size measurement of 0.2, with a significance level α = 0.05 and a power 1 − β = 0.8, for the Friedman’s test (three groups of repeated measurements).

## 3. Results

The study lot consisted of 90 individuals diagnosed with HCV infection in different stages of the disease (F2 = 3, F3 = 34, F4 = 53), recruited from those who received therapy with DAA for the 12 weeks within the Romanian National Protocol for HCV infected patients. The descriptive analysis of the demographic data has shown that 76.63% were women, and 53.3% of all patients had urban residency. According to their age, 13.33% were in the 30–55 years age group, 45.56% in the 56–65 years age group, and 41.11% in the 66–80 years age group. Age groups were similar in terms of gender and residency distribution, and no statistical differences were identified between these categories.

In what regards the proposed research, we have evaluated the level of perceived stress (PSS) for all subjects at the BSL, EOT, and SVR. We observed a predominance of the moderate level at all moments of evaluation (Table 2). Variations are mainly within the groups with low and high levels of PSS.

**Figure 1:**
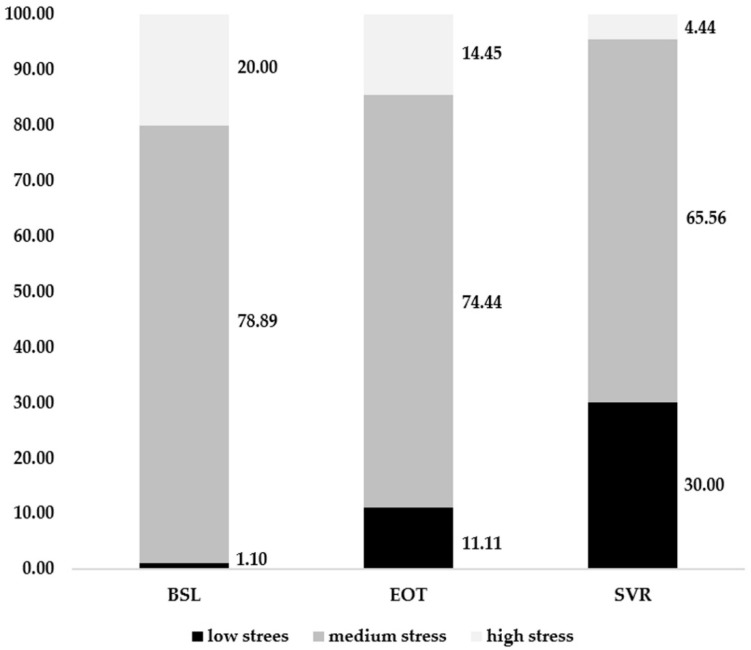
Evolution of PSS scores at BSL, EOT and SVR.

During the 12 weeks of treatment, between BSL and EOT, and 12 weeks of follow-up, between EOT and SVR, the overall number of patients with severe PSS levels presented mainly a descending tendency for all categories of patients, mostly in association with an increasing trend of patients with low PSS (Figure 1). Taking this decreasing trend into account, the evolution of the perceived stress during the treatment and the follow-up period was assessed and compared between BSL and EOT PSS scores, respectively, between EOT and SVR PSS scores. Thus, a positive difference represented a decrease in the PSS score, and a negative difference represented an increase in PSS score.

**Figure 2:**
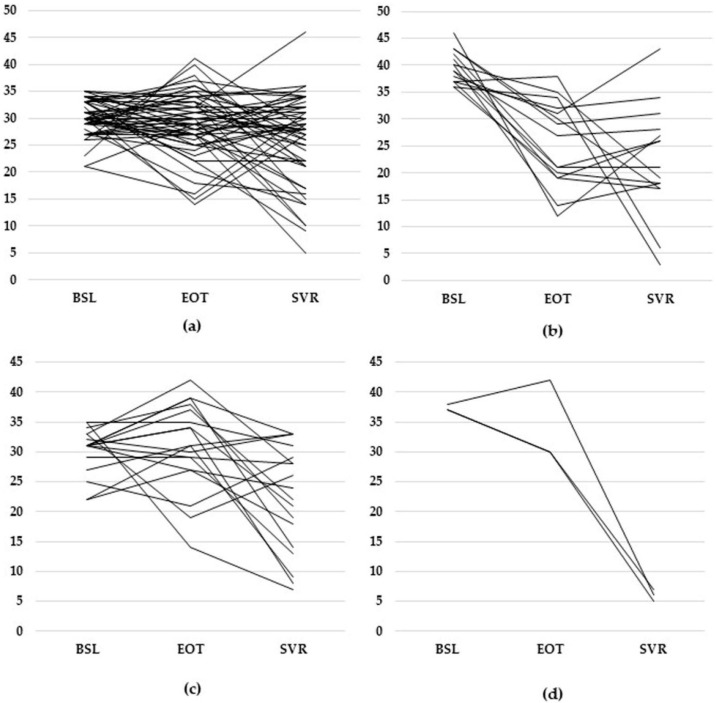
Evolution of individual PSS scores at BSL, EOT, and SVR: (**a**) female subjects with moderate stress levels at the beginning of the study, (**b**) female subjects with high stress levels at the beginning of the study, (**c**) male subjects with moderate stress levels at the beginning of the study, (**d**) male subjects with moderate stress levels at the beginning of the study (only one subject, a woman, had a low level of stress at BSL).

The analysis of PSS scores’ evolution between BSL and EOT revealed statistically significant differences between males and females (*p* = 0.034). The number of patients with low PSS increased in both groups; however, the variation in the high PSS level was opposite: decreased for females, increased for males (Figure 2). The analysis of the scores’ evolution at SVR vs. EOT revealed, once more, differences which were statistically significant (*p* = 0.003). This time, the percentage of males with low PSS levels was almost twice that compared to the females (Table 3).

The influence of the patients’ residence over the stress perception at BSL vs. EOT was also statistically significant (*p* = 0.036), with an increased number of urban patients being more relaxed at EOT. The stress–residence relationship as assessed between EOT and SVR was not statistically significant (*p* > 0.05) (Table 3).

The age of the subjects did not prove to be a significant factor to the influence on the evolution of stress’ perception during the study period or the follow-up period (*p* > 0.05), and neither for the distribution by age group (Table 3).

The clinical data of the study sample have shown that 45.56% of the subjects were overweight, according to the BMI value (25–29.9), while 27.78% presented obesity (class I and II). The weight factor had no statistically significant influence on the evolution of the perceived stress between BSL and EOT (τb = −0.088, *p* = 0.231) or EOT and SVR (τb = −0.046, *p* = 0.525) for the overall study group. For the treatment period, a Kruskal-Wallis H test was run to determine if there were differences in PSS scores between BMI groups that differed in their score evolution. Median differences of PSS scores increased from groups with normal weight (−1), to overweight (2), to obesity class I (2.5), to obesity class II (4) groups, but the differences between groups were not statistically significant. For EOT vs. SVR score evolution, the same test revealed median values did not indicate a linear trend between groups, and the corresponding differences were not statistically significant (Table 4).

The damage of the liver, as described by the fibrosis degree, was predominantly F4 (liver cirrhosis), in 58.89% of the subjects, followed by F3 degree, in 37.78%. The higher degree of the gravity of the liver infection could be considered one of the most important stressors during the whole evolution of the disease, leading to moderate and high perceived stress, even if the psychological impact decreased during the period of evaluation. A Kendall’s tau-b correlation test was used to analyze the relationship between the fibrosis degree and the overall PSS scores evolution. For the treatment period, there was a weak, positive association between these variables, which was not statistically significant, τb = 0.115, *p* = 0.188. Similar results were obtained for the follow-up period (τb = 0.043, *p* = 0.618) for the overall study group. The analysis of median values divided by groups with different scores revealed an increasing trend of the PSS score differences during the treatment period: the F2 group is characterized by the lowest median value (−5), which indicates increased stress value at the end of the treatment; the F3 group displays the median value of 0, indicating a relatively stable overall stress perception for this group during the treatment; the F4 group has the highest median value (2), reflecting an overall decreased stress score after the treatment. However, differences between groups were not statistically significant (Table 4). The follow-up analysis reflected only positive median values, and thus decreased stress levels for all groups at the end of the follow-up, but still with no statistically significant differences between groups with various F scores (Table 4).

The laboratory data relevant for the purposes of our study were HCV- RNA and AFP, which had the following mean values (±standard deviation) measured at the beginning of our study period (BSL): 1,178,736 ± 1,387,127 IU/mL, respectively 11.05 ± 16.45 IU/mL. Furthermore, we collected the values of the ALT (105.25 ± 86.99 IU/L) and AST (86.11 ± 55.79 IU/L). A Kendall’s tau-b correlation was run to determine the relationship between the BSL value of PSS and HCV-RNA at the same moment, and it showed a weak positive association, not statistically significant: τb = 0.073, *p* = 0.320. The same not significant level of correlation was obtained both for ALT (τb = −0.037, *p* = 0.624) and AST (τb = −0.065, *p* = 0.389). Instead, the relationship between values of PSS and AFP of our participants revealed a very weak, positive association, which was statistically significant, τb = 0.163, *p* = 0.029.

A Spearman’s rank-order correlation was run to assess the relationship between the differences of the PSS scores at BSL and EOT, versus the ALT and AST’s values at the same moments. Preliminary analysis showed these relationships to be monotonic, as they were assessed by visual inspection of the scatterplots, but not statistically significant according to the tests: PSS vs. ALT, rs (88) = 0.144, *p* = 0.175; PSS vs. AST, rs (88) = 0.033, *p* = 0.756. It kept the statistically significant level of correlation between PSS scores and AFP values at the beginning and end of the DAAs therapy (rs (88) = −0.239, *p* = 0.023). Regarding the possible associations linking the levels of stress and biochemical data at the EOT and SVR, the Kendall’s tau-b correlation test did not reveal statistically significant values (PSS vs. ALT: τb = −0.120, *p* = 0.098; PSS vs. AST: τb = −0.093, *p* = 0.200).

The complete analysis of the entire study period (treatment and follow-up) was based on a Friedman test used to determine whether there were differences in stress levels during the 24-week period. Pairwise comparisons (between the three different moments of time—BSL, EOT, and SVR) were performed in SPSS with a Bonferroni correction for multiple comparisons. The gender of the patients had a statistically significant influence over the perceived stress, from the beginning of the DAAs therapy (BSL) to the last moment of the evaluation (SVR), both on the Friedman test and the pairwise comparison. More than that, it could be observed during the treatment’s period (BSL to EOT) that the patient’s gender was not a factor of influence on their emotional status. During the whole study’s period (BSL to SVR), only the urban area had a significant impact on the perceived stress, a similar situation being noticed for the 56–65 age group. Out of the clinical status data recorded, the overweight and obesity class II influenced the perceived stress, respectively F4 degree of fibrosis (Table 5).

## 4. Discussion

The Hepatitis C Virus infection represents the background for disturbed psychological status and leads to higher levels of stress, as well as depression, anxiety, decreased quality of life, and affected social, financial, sexual, and family life. These facts have their origin even in the first moment of the diagnosis, inducing a considerable emotional and psychological burden [10,28]. When it comes to assessing the perceived stress level of the individuals with HCV infection, an important point was related to the delimitation between the effects of the stress generated by the discovery of the viral infection and the direct effects of the virus itself. In our case, the patients already had a history of HCV infection, so we evaluated only the effects of the disease and therapy on their emotional status. More than that, we did not intend to find the cerebral background of the induced stress on HCV infection patients, as other authors did [29,30]; we only aimed to find correlations between the level of stress and the positive outcomes of the DAA based therapy.

Since measurement of the initial perceived stress (BSL) has shown, in our study, a clear dominance of moderate and high levels of psychological disturbances, in the context of a higher frequency of severe liver disease (F3 and F4 degree of fibrosis), we could consider the situation was different than the one presented by other studies, where the emotional impairment was not significantly related to the histological severity of liver disease [31,32].

Our results have shown, during the DAAs treatment period (BSL to EOT), that the level of stress did not decrease in a significant way, the presence of the active disease remaining an important stressor and affecting patients’ quality of life, social activities, and physical conditions, as was noticed by other studies [10,33,34]. The most important observation resulting from our research was actually related to the significant decline in the severity of the stress in the period following the end of DAA based therapy to the SVR. Thus, we may state once the quantity of ARN-VHC became undetectable and the patient was aware of this, the direct effect on his/her psychological status was a significantly positive one, consistent with the results of previous research [35,36]. In this context, the importance of the increased patients’ health literacy and the necessity of improving their insight on the disease and the positive outcomes of the current therapeutic tools could be emphasized, in order to ensure the compliance to the new medication, but also to find the right coping mechanisms for the emotional distress.

Regarding the adherence to the DAAs therapy, the daily self-administration of the drugs may be considered less stressful compared to the previous interferon-based therapy in terms of duration of treatment (12 weeks vs. 48 weeks), method of administration (self-administered pills vs. weekly pen injection plus daily pills), and the number and complexity of the mandatory clinical and laboratory periodical investigations [37,38]. Knowledge about the efficacy of the DAAs could be highlighted, which, based on health literacy, personal and peers’ experiences, has led to an increased level of motivation following the prescribed therapy and lower levels of the perceived stress at the end of the treatment period. These assertions are consistent with previous studies which have shown skepticism about treatment effectiveness and tolerability, poor therapeutic relationship established between patient and the healthcare team, and other psychological personal conditions that could be expressed both in treatment failures and severe emotional impairments [39,40]. Moreover, the results of our study regarding the downward trend of the perceived stress associated with the HCV infection are comparable with other reports mentioning significantly lower levels of stress as long as liver diseases had decreased the impairment of daily life [41].

## 5. Conclusions

Psychological distress represents a serious barrier in the evolution and treatment of the HCV infection, and it is directly related to the disease, per se, and the therapeutic approach. For individuals with HCV infection treated with DAAs from our study sample, levels of perceived stress are significantly lower, both during the treatment and during the follow-up period. The factors related to this process are demographic (gender, residence, age), clinical (improvement of the liver disease), and biochemical (returned to normal values). The reduction in stress could be thus considered a valued benefit for the patients.

## Figures and Tables

**Table 1:** Inclusion and exclusion criteria for the Romanian national program of interferon-free therapy and our study.

**Romanian National Program of Interferon-Free Therapy**	
**Inclusion Criteria**	**Exclusion Criteria**
fibrosis F3/F4 METAVIR;	decompensated cirrhosis (Child-Pugh > 6);
fibrosis F0-F2 METAVIR only for healthcare professionals;	liver cancers without transplant indication;
HCV-HIV or HCV-HBV coinfection;	liver cancers ablatively treated or resected less than 6 months after the intervention;
hepatocellular carcinoma (HCC);	liver cancers with post-surgery CT/MRI signs of activity/recurrence;
post-transplant patients (other than liver);	contraindications to Ombitasvirum + Paritaprevirum + Ritonavirum or Dasabuvirum.
patients with extrahepatic malignancies;	
patients with hematological neoplasms.	
**Our Study**	
**Inclusion Criteria**	**Exclusion Criteria**
to be included in Romanian national program of interferon-free therapy;	refusal to participate in the study.
without neurologic and/or psychiatric disorder in the last 12 months.	

**Table 2:** The levels of perceived stress (PSS) within the study sample.

Group	Stage	PSS Score Level		
		Low	Moderate	High
Study lot *n* (%)	BSL	1 (1.11)	71 (78.89)	18 (20.00)
	EOT	10 (11.11)	67 (74.44)	13 (14.44)
	SVR	27 (30.00)	59 (65.56)	4 (4.44)

**Table 3:** The levels of perceived stress (PSS) correlated with the demographic characteristics of the study sample.

Group	Stage	PSS Score Level			BSL/EOT	EOT/SVR
		Low	Moderate	High		
Urban *n* (%)	BSL	0 (0.00)	33 (78.57)	9 (21.43)	U = 1267.5 z = 2.102 ***p*** ^1^ **= 0.036**	U = 1082 z = 0.599 *p* ^1^ = 0.549
	EOT	9 (21.43)	27 (64.29)	6 (14.29)		
	SVR	18 (42.86)	23 (54.76)	1 (2.08)		
Rural *n* (%)	BSL	1 (2.08)	38 (79.17)	9 (18.75)		
	EOT	1 (2.08)	40 (83.33)	7 (14.58)		
	SVR	9 (18.75)	36 (75.00)	3 (6.25)		
Female *n* (%)	BSL	1 (1.45)	53 (76.81)	15 (21.74)	U = 502.5 z = −2.121 ***p*** ^1^ **= 0.034**	U = 1032.5 z = 2.941 ***p*** ^1^ **= 0.003**
	EOT	8 (11.59)	54 (78.26)	7 (10.14)		
	SVR	17 (24.64)	48 (69.57)	4 (5.80)		
Male *n* (%)	BSL	0 (0.00)	18 (85.71)	3 (14.29)		
	EOT	2 (9.52)	13 (61.90)	6 (28.57)		
	SVR	10 (47.62)	11 (52.38)	0 (0.00)		
35–55 *n* (%)	BSL	0 (0.00)	7 (58.33)	5 (41.67)	χ^2^(2) = 0.301 *p* ^2^ = 0.860	χ^2^(2) = 0.654 *p* ^2^ = 0.721
	EOT	0 (0.00)	9 (75.00)	3 (25.00)		
	SVR	4 (33.33)	7 (58.33)	1 (8.34)		
56–65 *n* (%)	BSL	0 (0.00)	35 (85.37)	6 (14.63)		
	EOT	5 (12.20)	33 (80.49)	3 (7.32)		
	SVR	14 (34.14)	24 (58.54)	3 (7.32)		
66–80 *n* (%)	BSL	1 (2.70)	29 (78.38)	7 (18.92)		
	EOT	5 (13.51)	25 (67.57)	7 (18.92)		
	SVR	9 (24.32)	28 (75.68)	0 (0.00)		

^1^ Mann–Whitney U test; ^2^ Kruskal-Wallis H test.

**Table 4:** The levels of perceived stress (PSS) correlated with the clinical characteristics of the study sample.

Group	Stage	PSS Score Level			BSL/EOT	EOT/SVR
		Low	Moderate	High		
17–24.9 *n* (%)	BSL	1 (4.17)	17 (70.83)	6 (25.00)	χ^2^(3) = 2.798 *p* ^1^ = 0.424	χ^2^(3) = 3.347 *p* ^1^ = 0.341
	EOT	2 (8.33)	15 (62.50)	7 (29.17)		
	SVR	5 (20.83)	18 (75.00)	1 (4.17)		
25–29.9 *n* (%)	BSL	0 (0.00)	33 (80.49)	8 (19.51)		
	EOT	2 (4.88)	35 (85.37)	4 (9.76)		
	SVR	16 (39.02)	23 (56.10)	2 (4.88)		
30–34.9 *n* (%)	BSL	0 (0.00)	17 (85.00)	3 (15.00)		
	EOT	5 (25.00)	13 (65.00)	2 (10.00)		
	SVR	3 (15.00)	16 (80.00)	1 (5.00)		
35–39.9 *n* (%)	BSL	0 (0.00)	4 (80.00)	1 (20.00)		
	EOT	1 (20.00)	4 (80.00)	0 (0.00)		
	SVR	3 (60.00)	2 (40.00)	0 (0.00)		
F2 *n* (%)	BSL	0 (0.00)	3 (100.00)	0 (0.00)	χ^2^(2) = 2.202 *p* ^1^ = 0.333	χ^2^(2) = 0.908 *p* ^1^ = 0.635
	EOT	0 (0.00)	3 (100.00)	0 (0.00)		
	SVR	1 (33.33)	2 (66.67)	0 (0.00)		
F3 *n* (%)	BSL	0 (0.00)	29 (85.29)	5 (14.71)		
	EOT	4 (11.76)	23 (67.65)	7 (20.59)		
	SVR	9 (26.47)	23 (67.65)	2 (5.88)		
F4 *n* (%)	BSL	1 (1.89)	39 (73.58)	13 (24.53)		
	EOT	6 (11.32)	41 (77.36)	6 (11.32)		
	SVR	17 (32.08)	34 (64.15)	2 (3.77)		

^1^ Kruskal-Wallis H test.

**Table 5:** Statistical analysis of the study data (Friedman test, pairwise comparisons, median values).

		Friedman	Pairwise Comparisons (*p*)			Median PSS Score		
		(χ^2^, *p*)	BSL-EOT	BSL-SVR	EOT-SVR	BSL	EOT	SVR
Gender	Female	10.587; ***p*** **= 0.005**	0.090	**0.007**	1.000	31	29	27
	Male	10.927; ***p*** **= 0.004**	1	**0.041**	**0.006**	31	31	21
Residence	Rural	3.326; *p* = 0.190	n/a	n/a	n/a	31	30	28
	Urban	16.738; ***p*** **< 0.0005**	0.076	**<0.0005**	0.243	33	29.5	22
Age group (years)	30–55	2.913; ***p*** **= 0.023**	n/a	n/a	n/a	34.5	31	26.5
	56–65	9.713; ***p*** **= 0.008**	1	**0.015**	**0.039**	31	30	25
	66–80	5.348; ***p*** **= 0.069**	n/a	n/a	n/a	31	29	28
BMI (kg/m^2^)	17–24.9	1.780; *p* = 0.411	n/a	n/a	n/a	32	30	28
	25–29.9	9.686; ***p*** **= 0.008**	0.739	**0.009**	0.205	31	30	25
	30–34.9	4.430; *p* = 0.109	n/a	n/a	n/a	31	29	27.5
	35–39.9	7.444; ***p*** **= 0.024**	0.246	**0.034**	1	33	27	17
Fibrosis degree	F2	0.667; *p* = 0.717	n/a	n/a	n/a	29	31	28
	F3	2.800; *p* = 0.247	n/a	n/a	n/a	30	29.5	28
	F4	14.539; *** p* = 0.001**	0.156	**0.001**	0.217	33	30	26

n/a—not available.

## Data Availability

The data used in this study could be available by request and after the approval of the local IRB.
